# Supplementary material for: Towards Continuing Interprofessional Education: Interaction patterns of health professionals in a resource-limited setting
Source: PLoS One. 2021 Jul 9;16(7):e0253491. doi: 10.1371/journal.pone.0253491 (PMC8270436; doi:10.1371/journal.pone.0253491)
Supplement: S1 File — (DOCX) [file pone.0253491.s001.docx]

Hospital: X academic hospital, Bloemfontein

Ward: Paediatric ward

Preamble

The researchers arrive at the ward before the ward round

| Time | Observation | Reflection of the observer |
| --- | --- | --- |
| 740  955 | Focus: Round  Role players ( All members of the health team)  A team of doctors and medical students are in a cubicle. The doctor requests that the nurses should come through to the round. Nurses seem occupied and disappear in the other wards.  (the observations are withheld as this round does not meet the criteria for inclusion)  The round continues without the nursing professionals. There is confusion on one of the patients who is supposed to receive drug X. The doctor calls on a nurse to explain what is happening to the baby and the patient. The nurse is not sure what is going on and asks to be given time to go and inquire. She leaves the ward and returns after 4 mins to the same round. At the round the nurse stands at the back and as she explains she is hardly heard by the doctors until the senior doctor moves towards. The two, nurse and doctor, discuss the process, and the nurse explains how nurse gave the drug through alternative means. The doctor agrees on the method of administration. Immediately the nurse leaves the round and stands and chats with another nurse by the corridor.  (The researchers had been promised a typical interprofessional ward round- although they had to learning  The researchers wait for 2 hours for the round.  The consultant leading the round is apparently on leave and the ward round in cancelled. |  |
|  | New Cubicle  Role players: 2 Doctors and Nurses  Doctors still around the patient with nurses. The doctor reaches for the patient files and the nurse, still has their hands in their pockets. Doctors are talking to each other regarding a specific medical procedure for the patient. One of the doctors asks the nurse about the specific procedure for the patient, with which she nodes. They all laugh at a joke.  The one doctor continues writing on the notes, and on the side the nurse and the doctor continue with a social discussion and the patient continues to play with her baby.  The baby of the patients starts crying seriously, and the doctor orders the mother to deal with her baby as he is trying to work and the baby is making noise. At the same time the nurse, finds a chair and sits quietly, while the doctors now discuss the patient.  The doctors continue to discuss the treatment options and the nurse eventually starts playing with the baby and the baby stops crying. The doctors turn to the mother of the baby for her to respond to a series of questions. The mother is asked questions and she responds with one word answers as she is focusing on her phone.  The nurse seems not sure about the prescription, and asks an assistant nurse to explain the prescription to the mother. At that moment the assistant nurse nodes that “she will do it” but does not explain it.  The doctors wash their hands and engages with an examination of the baby.  The first doctor needs a confirmation from the second doctor on their physical examination findings. The second doctor immediately handles the patient without washing his hands. They seem to be in agreement with their findings, while the nurse is now looking out through the window.  A dietician comes to in to the cubicle and walks straight to the doctors to ask specifically about the management of a specific patient. She immediately leaves when is answered.  The patient talks to the nurse about a specific incidence that influenced the care of the baby.  The dietician walks in again, and talks to one of the patients in the cubicle. They keep their conversation private and she does not share with the other professionals in the team about their conversations and findings.  The nurse and the two doctors remained around the patient, and the doctor continues writing. The doctors tell the mother that she cannot go home as previously planned. The mother bursts out crying and sobs bitterly. The nurses clicks her pen continuously and engages in a conversation with the doctors. The patient continues to cry. The nurse asks the patient why she is crying and she explains her reason, the doctor walks away and the nurse starts to explain the rational for not discharging the baby.  The mother is still crying and not convinced with the discussion they have with the nurse. |  |
|  |  | |
| 945 |  |  |
