# Supplementary material for: Towards Continuing Interprofessional Education: Interaction patterns of health professionals in a resource-limited setting
Source: PLoS One. 2021 Jul 9;16(7):e0253491. doi: 10.1371/journal.pone.0253491 (PMC8270436; doi:10.1371/journal.pone.0253491)
Supplement: S2 File — (DOCX) [file pone.0253491.s002.docx]

|  | New Cubicle  Role players: 2 Doctors and Nurses  Doctors still around the patient with nurses. The doctor reaches for the patient files and the nurse, still has their hands in their pockets. Doctors are talking to each other regarding a specific medical procedure for the patient. One of the doctors asks the nurse about the specific procedure for the patient, with which she nodes. They all laugh at a joke.  The one doctor continues writing on the notes, and on the side the nurse and the doctor continue with a social discussion and the patient continues to play with her baby.  The baby of the patients starts crying seriously, and the doctor orders the mother to deal with her baby as he is trying to work and the baby is making noise. At the same time the nurse, finds a chair and sits quietly, while the doctors now discuss the patient.  The doctors continue to discuss the treatment options and the nurse eventually starts playing with the baby and the baby stops crying. The doctors turn to the mother of the baby for her to respond to a series of questions. The mother is asked questions and she responds with one word answers as she is focusing on her phone.  The nurse seems not sure about the prescription, and asks an assistant nurse to explain the prescription to the mother. At that moment the assistant nurse nodes that “she will do it” but does not explain it.  The doctors wash their hands and engages with an examination of the baby.  The first doctor needs a confirmation from the second doctor on their physical examination findings. The second doctor immediately handles the patient without washing his hands. They seem to be in agreement with their findings, while the nurse is now looking out through the window.  A dietician comes to in to the cubicle and walks straight to the doctors to ask specifically about the management of a specific patient. She immediately leaves when is answered.  The patient talks to the nurse about a specific incidence that influenced the care of the baby.  The dietician walks in again, and talks to one of the patients in the cubicle. They keep their conversation private and she does not share with the other professionals in the team about their conversations and findings.  The nurse and the two doctors remained around the patient, and the doctor continues writing. The doctors tell the mother that she cannot go home as previously planned. The mother bursts out crying and sobs bitterly. The nurses clicks her pen continuously and engages in a conversation with the doctors. The patient continues to cry. The nurse asks the patient why she is crying and she explains her reason, the doctor walks away and the nurse starts to explain the rational for not discharging the baby.  The mother is still crying and not convinced with the discussion they have with the nurse.  The doctor moves swiftly to the next patient while the other mother is still crying. A second nurse joins the round after taking note of the crying of the mother of the other baby. The initial nurse continues clicking her pen and the second nurses puts her hands in her pockets and is ready to leave the round. The doctor explains that there will be surgery for the baby and that the patient needs to sign a consent form. As the doctor discusses the consent with the patient, the nurse pulls a chair in the round and finds a place to sit comfortably. The patient is made to sign the consent form regarding a surgical procedure for the baby. | The doctor does not explain to the mother what and why is it happening.  The nurse is very rude and literally talks down to the patient, even suggesting that she can go if she wants and leaves the baby behind in the hospital, since she is “so eager to go” |
| --- | --- | --- |
|  |  |  |
|  |  |  |
