# Supplementary material for: Towards Continuing Interprofessional Education: Interaction patterns of health professionals in a resource-limited setting
Source: PLoS One. 2021 Jul 9;16(7):e0253491. doi: 10.1371/journal.pone.0253491 (PMC8270436; doi:10.1371/journal.pone.0253491)
Supplement: S3 File — (DOCX) [file pone.0253491.s003.docx]

Hospital: X academic hospital, Bloemfontein

Ward: Paediatric ward

Preamble

The researchers arrive at 10 am in the hospital after a hospital round.

Day 2: Observation

Role players: Nurse, students and Patients

| Time | Observation | Reflection of the observer |
| --- | --- | --- |
|  | Focus: Nursing cleaning wound  The nurse cleans the wound and explains to the patient on what should happen to the wound when they reach home. A nursing student looks on, and provides a few supplies as the nurse continues cleaning the wound. There is a brief argument between the nurse and the student on how the wound should dressed. The nurse eventually explains the purpose and the procedure to the student and the reasoning behind the procedure.  The mother asks a question about the wound and the baby, the nurse responds appropriately, and the baby talks back to the nurse. | This is a ward, which include some cancer patient who are constantly having wound dressings. The babies in this ward are screaming in pain due to the wound dressing. |
|  | Opposite the first patient, the consultant/doctor arrives and quickly asks for patient charts. He is asking for patient charts in “his” ward.  As soon as the consultant asks for the patient chart. The physiotherapist who was engaged with the care of one of the patients in the cubicle leaves.  The physiotherapist returns after a few minutes to give a report to the doctor on what she has been doing to the patient, and she expresses a specific concern. They both leave the room. Left in the room is the nurse and the patient, and the nurse seems clueless on the discussions of the doctor and the physiotherapist.  The physiotherapist and doctor have left the room, leaving the nurse alone. | The mention for the need of patient charts in his ward is interesting to the observer.  The nurse seems not interested to be included in the discussion between the doctor and the physiotherapist. She continues writing on her notes |
| 920 | Focus: A specific clinical procedure is being done on the baby. Professionals present include nurse and doctor and then there is the mother of the baby.  The doctor is directly engaged with a specific procedure with the baby. The nurse leaves the room, leaving the mothers and doctor. | Setting: A cubicle with four beds, 4 babies and their mothers and a crying baby.  There is limited interaction and communication between the doctor and the nurse. |
| 945 | Focus: A patient is about to be discharged  Role players: Doctor, Nurse and nursing students  A doctor is seen on the trolley and are continuously writing with no communication with the other members of the team. The doctor asks the patient about specific medication history and the patient seems not sure. The nurse does not intervene, as she seems not to be sure of the patient medication as well. The doctor continues writing.  After the doctor has completed writing, he closes the file and leaves the cubicle. The nurses in the wards then reach for the file and read the prescription and records on a different file.  The doctor returns back to the cubicle, asks for the same file and further makes a recording. He then leaves | The researchers arrive when the patient has been discharged and a discharge summary is being constructed.  The communication between health professionals is between files and not verbal.  Observation ends at 10am |
|  | Reflections of the day  Reflections by the researchers: CN, CG, YB  What: Limited or not interaction between professionals during a procedure  Doctors, nurses, physiotherapist not talking to each other  There is no consultation on the findings of each professional  Independence and working in “silo” on one patient  So what: There is a gap in the communication between professionals in the hospital. The nurses seems absent from the patient care most of the time, with their concentration on the corridors. “everyone seems to be in the hall ways, and not with the patients”. There seem to be no clear model of care for professions in the ward especially for nurses.  What next | |
|  |  |  |
|  |  |  |
