# Supplementary material for: Towards Continuing Interprofessional Education: Interaction patterns of health professionals in a resource-limited setting
Source: PLoS One. 2021 Jul 9;16(7):e0253491. doi: 10.1371/journal.pone.0253491 (PMC8270436; doi:10.1371/journal.pone.0253491)
Supplement: S4 File — (DOCX) [file pone.0253491.s004.docx]

Hospital: X academic hospital, Bloemfontein

Ward: Paediatric ward

Preamble

The researchers arrive at the ward before the ward round

| Time | Observation | Reflection of the observer |
| --- | --- | --- |
| 824 | Focus: Ward Round  Role players: patients  Doctor arrives for the ward round. The setting is within a cubicle. The doctor engages the nurse on the patient. The nurse translates to the patient what the doctor was saying. As the nurse translates to the patient, the doctor reaches for the notes of the patient and seems not impressed. The doctor leaves the room leaving the patient and the sister. The doctor exclaims that he is looking for a form for the patient.  The doctor returns after a while and prescribes specific treatment for the baby, and describes an instruction to the nurse. At around the same time the baby starts to cry, the nurses gives her back to the patient that she listens carefully to the doctor’s message. The baby continues crying.  A second doctor enters the scene and gets holds of the patient’s charts. The second doctor confirms with the first doctor on the treatment plan. The secretary of the unit, enters the scene for a “quick” confirmation with the doctors. The doctors gives a stern instruction for his orders to be followed, insinuating that he has been making specific patient orders and the nurses and mother of the patient have not been following them. At the same time, the mother asks a question to the team and no one responds. The nurse still giving her back to the mother, the nurses shadows the patient obstructs the patient and the doctor.  The Doctor explains to the mother that they will take out the drip and the antibiotics for the baby. The doctor asks if the mother has any specific questions. She says “no”.  A discussion between the doctors continue, and the nurses join in. The conversations is social and has no bearing with the patient. The one doctor washes his hands to examine the baby.  Without talking to the mother, holds the baby and examines it. The baby is crying continuously, the Doctor asks several questions to the mother in her own language. The mother responds to the doctor and they have a brief discussion about the baby, but he does not explain what he was examining on the baby nor his findings from such an examination.  After the examination, the doctor leaves the patient and washes his hands. After this, they round moves to the next patient to remove an NGT. The nurse follows closely, and keeps her hands in her pockets. After the NGT is removed, the mother does not receive any specific information regarding her future treatment. The nurse, who is now standing in the corner of the cubicle, asks a question about the patient’s X ray and she receives no response.  The nurse then approaches the patient who had an NGT removed to check on the baby, while the doctor examines the baby thoroughly.  Dr gives a summary of what has happened to the mother including, why he has removed the NGT and what should happen next. The doctor then concludes the rounds.  The nurse recommends to the doctors for the patients to be engaged with stoma care, and they all agree and document the recommendation on the charts. | The patient care lies heavily on the mother. The round is dependent on the arrival of a specific doctor.  The communication between doctors and nurses is poor, it seems the doctors don’t know what information nurses should or can provide, while nurses take a space at following the doctors.  Its interesting how when the patient is confronted may actually not have specific information to ask… in as much as a few lines ago she asked questions.  The patient seems more appreciative of being communicated on in their own language  It was noted that summaries were given to the patients and not the nurses |
|  |  |  |
|  | Reflection   - Hierarchy among professionals still present “Doctors speak and nurses listen” - Nurses in change have limited involvement in ward rounds - There is some interaction between doctors and the nurses, but such interactions are along the lines of the doctors telling the nurses what needs to be done - The size of the ward/cubicle is ideal for planning and collaboration - Patients not really involved in the care of the patient | |
| 945 |  |  |
