# Supplementary material for: Towards Continuing Interprofessional Education: Interaction patterns of health professionals in a resource-limited setting
Source: PLoS One. 2021 Jul 9;16(7):e0253491. doi: 10.1371/journal.pone.0253491 (PMC8270436; doi:10.1371/journal.pone.0253491)
Supplement: Attachment — Submitted filename: Response to reviewers comments.docx [file pone.0253491.s006.docx]

| Reviewer Comments | Author responses |
| --- | --- |
| Please tell the readers more about the resource-limited situation as mentioned in the title. We as the reader might learn more from your paper if we get the picture of the context that you are presenting | The authors have described the context of this study in the heading “context of the study” which generally describes the health delivery system in South Africa and also in the hospital included in this study. We have added an additional sentence to the manuscript to enhance clarity on the picture of the resource limitation of the study. |
| You have performed systematic three step analysis. Please tell the reader more about the process of how you got into the three themes as mentioned in the findings. Visual presentation eg a table or figure is recommended | The process of data analysis was described extensively under the section of data analysis including how each step of data analysis was executed including the coding methods for data analysis as supported by Saldana (2016). We have also added a sentence that explains pattern coding which underpinned the theme development process of this study. We are also conscious of the word count, limit and structure of the paper to add more. |
| Good luck | Thank you, your comments were insightful. |
